# Molecular Detection of Undeclared Porcine DNA in Processed Meat Products and Assessment of Halal Labelling Compliance in Kazakhstan Using qPCR

**DOI:** 10.3390/foods15213876

**Published:** 2026-10-29

**Authors:** Gaukhar Nusupbaeva, Damir Kobzhassarov, Sandugash Mukazhanova, Zhanat Zhamalbekova, Sandugash Katysheva, Aliya Ibrayeva, Raushan Olzhabek, Madina Tleubergenova, Saken Khaidarov, Aizhan Moldakaryzova, Perizat Omirali, Mukhit Kulmaganbetov

**Affiliations:** 1 Centre for Sanitary and Epidemiological Expertise of the Medical Centre of the Presidential Administration of the Republic of Kazakhstan, Astana 010020, Kazakhstan; 2 Department of Molecular Biology and Medical Genetics, Asfendiyarov Kazakh National Medical University, Almaty 050012, Kazakhstan; khaidarov.s@kaznmu.kz (S.K.);; 3 Alatau Assistance Medical Centre, Almaty 050043, Kazakhstan; 4 Centre for Eye and Vision Research, Hong Kong SAR, China

**Keywords:** meat adulteration, porcine DNA, qPCR, Halal certification, Kazakhstan, food safety, cross-contamination

## Abstract

Background: Meat adulteration, particularly the undeclared addition of pork, is a critical food fraud and religious compliance concern in Muslim-majority countries like Kazakhstan. This study determined the prevalence of undeclared porcine DNA in processed meat products and evaluated the reliability of Halal certification. Methods: A total of 195 non-pork-labelled samples collected across Kazakhstan in 2024–2025 were screened using species-specific quantitative PCR (qPCR). A Cycle Threshold (Ct) cutoff of 35 was applied to differentiate high target DNA concentrations from trace cross-contamination. Results: Undeclared porcine DNA was detected in 57 samples (29.2%). Of these, 48 (24.6%) yielded Ct values below 35, indicating high target DNA concentrations, while 9 (4.6%) had Ct values of 35 or above, suggesting trace cross-contamination. Notably, the detection rate in Halal-certified products (29.2%) was statistically identical to that of non-certified products (*p* = 1). Conclusions: These results reveal a substantial prevalence of undeclared porcine DNA in Kazakhstan’s processed meats and demonstrate that current voluntary Halal labelling provides no assurance against pork contamination. This underscores the urgent need for routine molecular surveillance and strict hygiene control protocols.

## 1. Introduction

Meat adulteration, the intentional or unintentional substitution, addition, or misrepresentation of meat species in food products, has become one of the most pervasive forms of food fraud globally [1,2,3]. The economic motivation for such practices is considerable: substituting expensive meats such as beef, horse, or turkey with lower-cost alternatives, particularly pork, can yield substantial profit margins for unscrupulous manufacturers [4,5]. Beyond the economic dimension, however, meat adulteration carries profound implications for public health, consumer trust, religious compliance, and international trade [1,6]. The adulteration of meat products is particularly sensitive in regions where religious dietary laws govern food consumption. In Muslim-majority societies, the consumption of pork is strictly prohibited by Islamic law (Halal), and the presence of undeclared porcine material in food products represents not merely a regulatory violation but a serious ethical and religious transgression [7].

Kazakhstan, where approximately 70% of the population identifies as Muslim, represents a critical yet understudied market for Halal food integrity [8]. The country’s processed meat industry has expanded significantly in recent years, driven by urbanisation, rising disposable incomes, and the increasing availability of processed meat products in retail chains. Despite this growth, regulatory frameworks governing meat species authentication and Halal certification remain relatively underdeveloped compared to those in the European Union or neighbouring countries [1]. The Halal certification process in Kazakhstan is currently managed by multiple independent bodies, creating potential vulnerabilities in the integrity of certification and enforcement.

Molecular methods, particularly Quantitative Polymerase Chain Reaction (qPCR), have become the gold standard for detecting and quantifying species-specific DNA in processed meat products [9,10]. Unlike protein-based methods such as enzyme-linked immunosorbent assay (ELISA), which may be compromised by protein denaturation during thermal processing [11], DNA-based techniques are robust across a wide range of processing conditions, including high-temperature treatments, curing, and smoking. qPCR offers the additional advantages [9] of high sensitivity, specificity, and the ability to provide quantitative information through Cycle Threshold (Ct) values [12], which can be used to estimate the relative amount of target DNA present in a sample [13]. This quantification capability is particularly valuable for distinguishing between high target DNA concentrations, which suggest the presence of substantial porcine tissue, and incidental cross-contamination, in which trace amounts may be introduced through shared processing equipment or inadequate cleaning protocols.

To date, no comprehensive molecular study has investigated the prevalence of undeclared porcine DNA in processed meat products in Kazakhstan. This study addresses this critical gap by screening 195 commercially available processed meat samples collected during 2024–2025 using species-specific qPCR targeting porcine DNA. The specific objectives were: (1) to determine the overall prevalence of undeclared porcine DNA in non-pork labelled processed meat products; (2) to differentiate between high target DNA concentration and trace cross-contamination using a Ct value threshold of 35; (3) to evaluate the consistency between Halal labelling and the absence of porcine DNA in the sampled products; and (4) to evaluate the effect of the level of processing on the likelihood of porcine DNA detection.

## 2. Materials and Methods

### 2.1. Sample Collection

A total of 201 processed meat product samples were systematically collected from retail outlets across Kazakhstan over a two-year period spanning 2024 (99 samples) and 2025 (102 samples). Sampling was designed to cover a broad spectrum of product types commonly available to consumers, including cooked sausages, semi-smoked sausages, dry sausages, sausages, sardelki, dumplings, canned meat, pates, and traditional Kazakh meat products (kazy, shuzhuk, basturma). Products were selected based on their declared meat species on the label, which included beef, horse meat, turkey, chicken, mutton, cheese-based products, unspecified meat products, and products with no species declaration. Six samples explicitly labelled as containing pork were excluded from the adulteration analysis, as the detection of porcine DNA in pork-labelled products does not constitute adulteration. Following exclusion, 195 samples were retained for statistical analysis.

Each sample was classified according to two additional variables: the level of processing (high processing: sausages, canned meat, pates, and other comminuted products; low processing: steaks, whole-muscle products, and minimally processed items) and Halal certification status. All samples were transported to the laboratory under refrigerated conditions (2–4 °C) and stored at −20 °C prior to analysis.

### 2.2. DNA Extraction

Genomic DNA was extracted from approximately 25 mg of homogenised meat tissue using a DNeasy Mericon Food Kit (Qiagen, Hilden, Germany) following the manufacturer’s recommended protocol, with minor modifications to enhance yield from processed matrices. Briefly, samples were lysed with Proteinase K at 56 °C, followed by column-based purification. The concentration and purity of the extracted DNA were assessed spectrophotometrically using a NanoDrop 2000 instrument (Thermo Fisher Scientific, Waltham, MA, USA). DNA samples with A260/A280 ratios between 1.7 and 1.9 were considered acceptable for downstream PCR amplification [14], serving as a preliminary indicator of DNA purity. However, acceptable absorbance ratios do not necessarily guarantee the absence of PCR inhibitors, as certain inhibitory substances in complex food matrices may not absorb light at the measured spectrophotometric wavelengths and can thus remain undetected. Extracted DNA was stored at −20 °C until use. DNA extraction was performed across 10 batches. qPCR analysis was conducted across 3 runs.

### 2.3. Quantitative PCR Analysis

Species-specific qPCR was performed using porcine-specific primers and probes targeting a conserved region of the mitochondrial cytochrome b (cyt b) gene [15] (Forward: 5′-CGGACATCCCAAAATCCA-3′; Reverse: 5′-CCCGATTCCACTCCAAC-3′; Probe: 5′-FAM-CCATTCACCCTCCTTT-BHQ1-3′).

To preserve assay sensitivity for trace targets and reflect the original tissue matrix composition, DNA input was normalised by tissue mass (25 mg) and elution volume rather than by standardising total DNA concentration across samples; a fixed volume of 5 μL of template extract was used per reaction [15,16]. Each 25 μL reaction mixture contained 12.5 μL of 2× TaqMan Universal Master Mix, 0.5 μM of each primer, 0.2 μM of the TaqMan probe, and a template DNA [15,16]. Amplification was conducted on a CFX96 Real-Time PCR Detection System (Bio-Rad Laboratories, Hercules, CA, USA) under the following cycling conditions: initial denaturation at 95 °C for 10 min, followed by 40 cycles of denaturation at 95 °C for 15 s and combined annealing/extension at 60 °C for 60 s [17].

The assay demonstrated an efficiency of 98.5% (y = −3.36x + 37.2, R^2^ = 0.999), with a limit of detection (LOD) established at 10 copies/µL using a 10-fold serial dilution of reference porcine DNA. In silico specificity was confirmed via Basic Local Alignment Search Tool (NCBI BLAST+ 2.17.0), and in vitro testing showed no cross-reactivity with beef, horse, chicken, turkey, or mutton DNA. All qPCR reactions were performed in technical triplicate, and a sample was considered positive if the target was detected in all replicates. Each run included a no-template control (NTC) and a positive control consisting of 10 ng/µL of DNA extracted from certified pork tissue, which consistently yielded a Ct value of 19.5 ± 0.3.

Samples yielding a quantification cycle (Ct) value within 40 cycles were classified as positive for porcine DNA [18]. A critical analytical threshold was established at Ct = 35 to differentiate between two scenarios: samples with Ct < 35 were classified as containing high concentrations of porcine DNA (indicative of significant tissue presence); samples with Ct ≥ 35 were classified as potential trace/cross-contamination events, representing trace amounts of porcine DNA [18,19,20] introduced through shared cutting boards, grinders, or inadequate sanitation protocols rather than deliberate substitution [21]. While this threshold is widely employed in meat authentication literature to empirically distinguish between bulk tissue and trace environmental carryover [18,19,20], it represents a pragmatic surveillance cut-off rather than a matrix-specific validated threshold for intent.

### 2.4. Statistical Analysis

The overall prevalence of undeclared porcine DNA was calculated as the proportion of positive samples among all non-pork-labelled products, with 95% confidence intervals (CI) computed using the Wilson score method, which provides more accurate interval estimates than the normal approximation for proportions, particularly when sample sizes are moderate [22]. Descriptive statistics (mean, standard deviation, median, range) were calculated for Ct values of positive samples.

Associations between porcine DNA detection and categorical variables (Halal certification status and processing level) were evaluated using Pearson’s Chi-square test with Yates’ continuity correction. When expected cell frequencies fell below 5, Fisher’s exact test was additionally computed as a robustness check. Odds ratios (OR) with 95% CIs were derived to quantify the strength of associations. All statistical analyses were performed using Python 3.11 with the SciPy statistical library (v1.11.0). A two-tailed significance level of *α* = 0.05 was adopted for all hypothesis tests.

## 3. Results

### 3.1. Overall Prevalence of Undeclared Porcine DNA

Of the 201 processed meat samples collected, in 195 non-pork-labelled samples (6 samples explicitly labelled as pork), undeclared porcine DNA was detected in 57 samples, yielding an overall prevalence of 29.2% (95% CI: 23.3–36%). The annual breakdown revealed comparable detection rates: 26 of 93 samples (28%) in 2024 and 31 of 102 samples (30.4%) in 2025.

**Figure 1:**
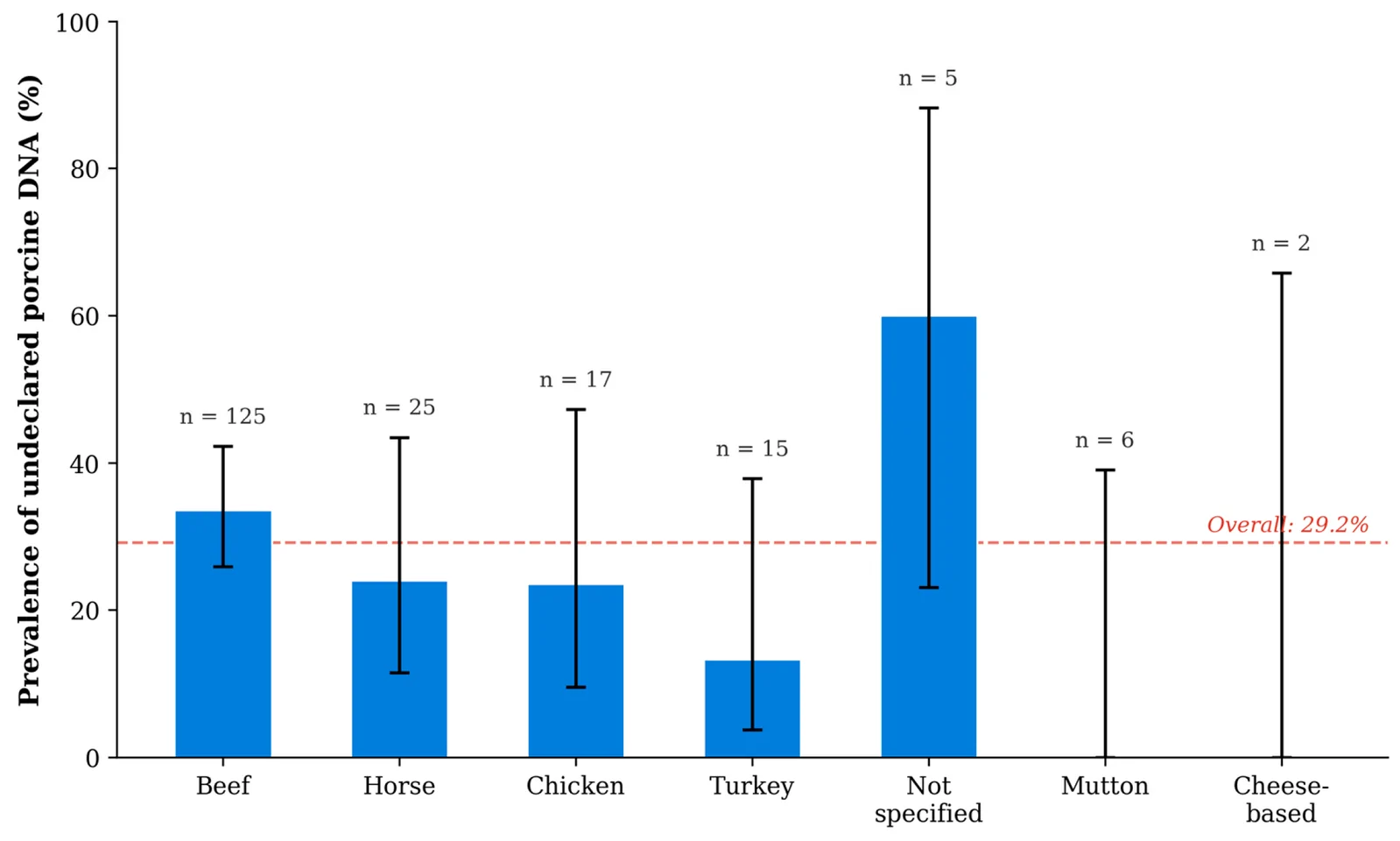
Prevalence of undeclared porcine DNA by product label category. Error bars represent 95% Wilson score confidence intervals. Sample sizes for each category are displayed above the bars.

The distribution of positive samples across product categories is summarised in Figure 1. Beef-labelled products accounted for the largest absolute number of positive samples (*n* = 42), consistent with their predominance in the sampling frame (*n* = 125). Notably, products with no specified meat type exhibited the highest proportional prevalence (60%, 3/5), though the small sample size warrants cautious interpretation. Among traditionally Halal-expected categories, horse meat products showed a 24% detection rate (6/25), while chicken and turkey products demonstrated rates of 23.5% (4/17) and 13.3% (2/15), respectively. Mutton products (*n* = 6) and cheese-based products (*n* = 2) showed no detectable porcine DNA.

### 3.2. Differentiation of High Target DNA Concentration and Trace Contamination

Application of the Ct = 35 threshold revealed that the vast majority of positive samples (48/57, 84.2%) yielded Ct values below 35, indicative of high target DNA levels [18,19,20]. Specifically, 48 samples (24.6% of all 195 non-pork samples) were classified as containing high target DNA levels, while 9 samples (4.6%) were classified as cross-contamination events based on Ct values ≥ 35. The mean Ct value across all 57 positive samples was 28.43 ± 4.9 (range: 13.65–37.65, median: 28).

**Figure 2:**
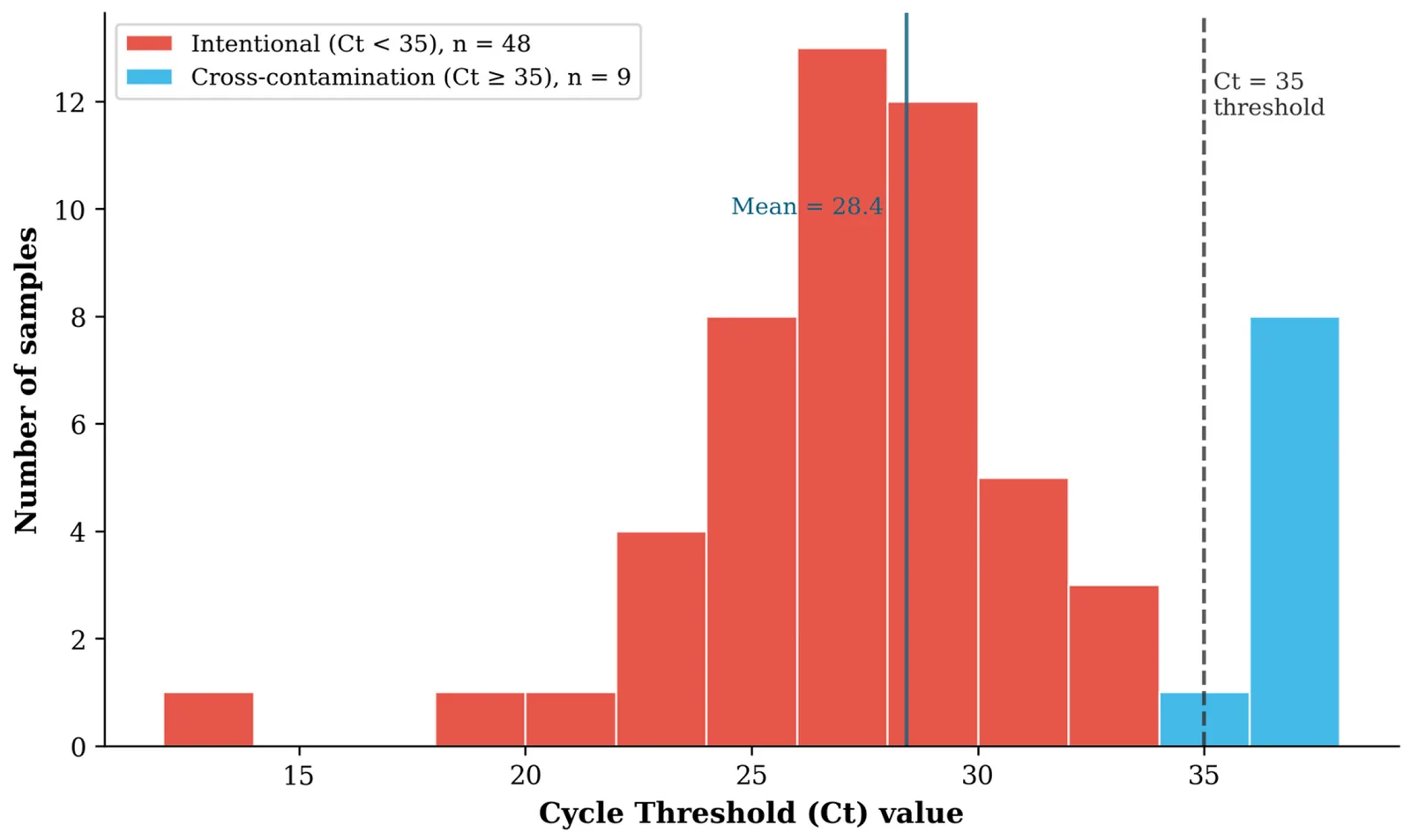
Distribution of Cycle Threshold (Ct) values among porcine DNA-positive samples (*n* = 57). The vertical dashed line indicates the Ct = 35 threshold used to differentiate high DNA concentration (Ct < 35, red) from cross-contamination (Ct ≥ 35, blue).

For samples with high target DNA concentrations (Ct < 35), the mean Ct was 26.85 ± 3.53, reflecting moderate to high concentrations of porcine DNA. In contrast, the 9 cross-contamination samples yielded a mean Ct of 36.85 ± 0.5, indicating substantially lower target DNA quantities. The distribution of Ct values is presented in Figure 2.

### 3.3. Association Between Halal Certification and Porcine DNA Detection

**Figure 3:**
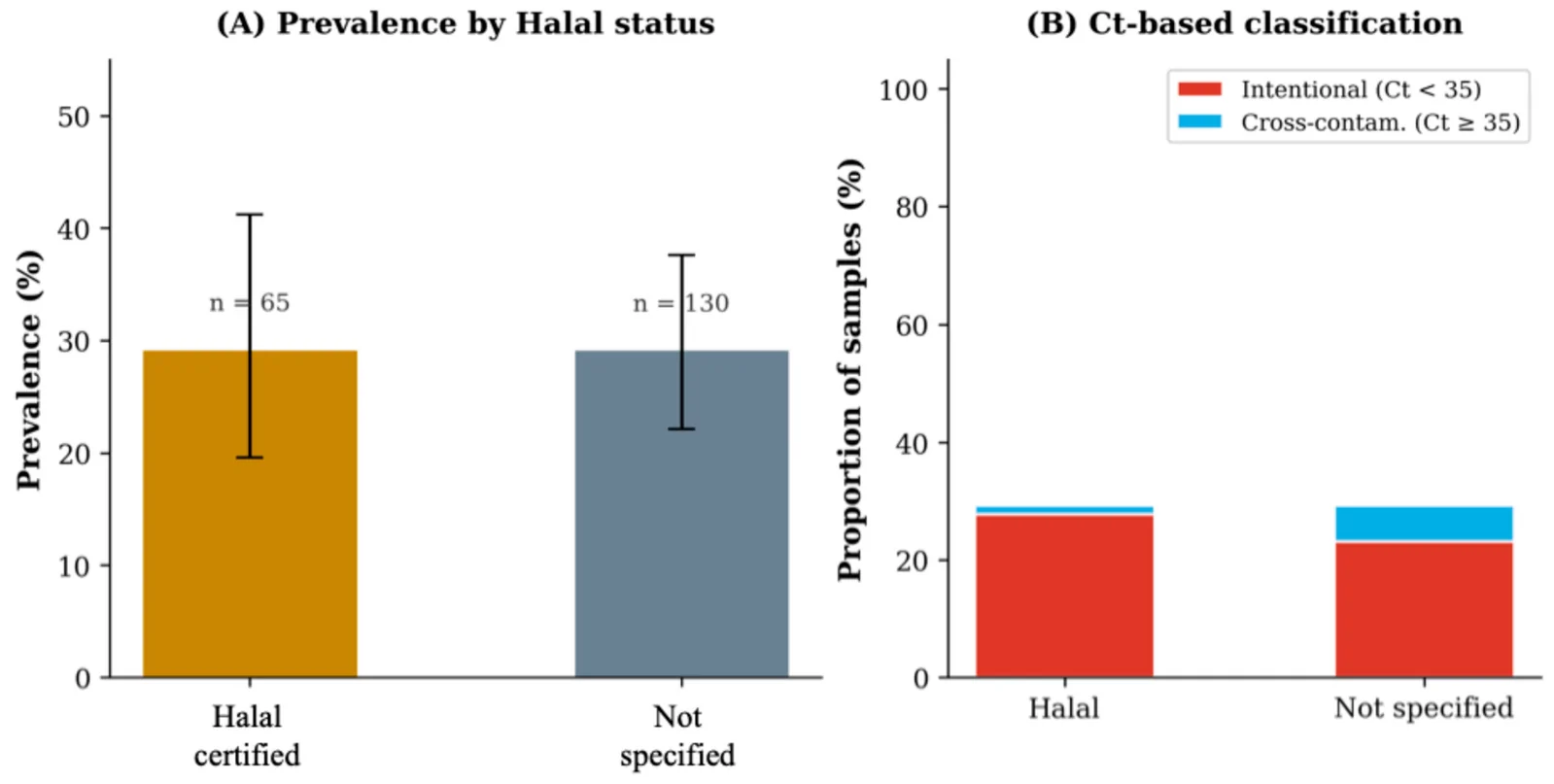
Comparison of porcine DNA detection between Halal-certified and non-certified products. (**A**) Prevalence with 95% Wilson score confidence intervals; (**B**) Proportion of samples classified as high DNA concentration (Ct < 35) versus cross-contamination (Ct ≥ 35).

The relationship between Halal certification and the presence of undeclared porcine DNA is presented in Figure 3. Among 65 Halal-certified products, 19 (29.2%) tested positive for porcine DNA, a rate virtually identical to that observed among 130 non-certified products, of which 38 (29.2%) were positive. The Chi-square test with Yates’ correction yielded *χ*^2^ = 0 (*p* = 1), and Fisher’s exact test confirmed this finding (OR = 1, *p* = 1). This indicates no association whatsoever between Halal labelling and the likelihood of porcine DNA detection.

Of particular concern, 18 of the 19 positive Halal-certified samples (94.7%) had Ct values below 35, classifying them as cases with high target DNA concentrations rather than trace cross-contamination. Only 1 Halal-positive sample yielded a Ct value ≥ 35 (36.85), suggesting cross-contamination. This finding indicates that the presence of porcine DNA in Halal-labelled products in the analysed sample set was predominantly associated with high target DNA concentrations rather than trace technological contamination alone.

### 3.4. Effect of Processing Level on Porcine DNA Detection

The association between the level of processing and porcine DNA detection is presented in Table 1. Highly processed products (sausages, canned meat, pates; *n* = 142) exhibited a numerically higher prevalence of porcine DNA (33.1%) compared to minimally processed products (steaks, whole-muscle products; *n* = 53, prevalence = 18.9%). However, this difference did not reach statistical significance using the Chi-square test with Yates’ correction (*χ*^2^ = 3.1217, *p* = 0.0773). Fisher’s exact test yielded a borderline result (OR = 2.1274, *p* = 0.0541), suggesting a potential trend that may warrant investigation with larger sample sizes. The full dataset of the study is presented as Supplementary Materials (Table S1).

The higher prevalence in highly processed products is biologically plausible, as comminuted and emulsified meat products offer greater opportunity for species substitution, as the original tissue morphology is destroyed during processing, making visual identification of adulteration impossible. Furthermore, the complex ingredient formulations of processed meats may mask the sensory characteristics of substituted species.

## 4. Discussion

The present study represents the first known molecular investigation of undeclared porcine DNA in processed meat products commercially available in Kazakhstan. The overall detection rate of 29.2% (95% CI: 23.3–36%) is alarming and positions Kazakhstan among the countries with the highest reported rates of porcine adulteration in non-pork meat products globally [1,23,24]. This finding is particularly concerning given the demographic composition of the country, where the majority of the population adheres to Islamic dietary laws that strictly prohibit pork consumption.

The observed prevalence is substantially higher than rates reported in several comparable studies conducted in other Muslim-majority and non-Muslim countries. In Turkey, a study by Ulca et al. (2013) reported porcine DNA in <0.1% of tested meat products [25], while in China, where pork consumption is culturally normative, but concerns about species substitution exist, Li et al. (2023) reported a porcine adulteration rate of ~15% in processed meat products [23]. European studies have generally reported lower rates: a multi-country survey by the European Commission following the 2013 horse meat scandal found less than 5% of tested products contained undeclared species [26].

The markedly higher rate observed in Kazakhstan may reflect several interconnected factors, including weaker regulatory enforcement, limited routine surveillance by food safety authorities, and the economic incentive for substitution in a market where price competition among domestic manufacturers is intense.

It is important to note that the beef-labelled category, which constituted the largest proportion of the sampling frame (64.1%), exhibited a 33.6% detection rate. This is particularly significant because beef is the most commonly consumed and trusted meat species in Kazakhstan, and consumers purchasing beef products have a reasonable expectation that these products contain exclusively bovine tissue. The high rate of porcine DNA detection in beef-labelled products suggests potential issues in supply chain integrity rather than isolated contamination events.

The application of a Ct = 35 threshold to differentiate between high target DNA concentrations and cross-contamination provides critical insight into the nature of porcine DNA presence in Kazakhstani meat products. The finding that 84.2% of positive samples fell below this threshold strongly indicates that the majority of positive samples contained substantial initial target DNA concentrations rather than incidental contamination. The mean Ct of 26.85 ± 3.53 for the high concentration group corresponds to a substantially higher initial porcine DNA template concentration, consistent with the addition of pork tissue as a meaningful ingredient rather than as a trace contaminant.

In contrast, the 9 samples classified as cross-contamination (Ct ≥ 35) exhibited a mean Ct of 36.85 ± 0.5, indicating very low copy numbers of porcine DNA. These trace-level detections are most plausibly explained by inadequate sanitation of shared processing equipment, such as grinders, stuffing machines, and cutting boards, between production runs handling different meat species [21,27,28]. This finding points to deficiencies in Good Manufacturing Practices (GMP) and Standard Sanitation Operating Procedures (SSOPs) at the production facilities sampled. While trace contamination may not reflect intentional addition, it nonetheless represents a significant compliance failure, particularly for Halal-certified products where any level of porcine contamination is unacceptable.

Perhaps the most concerning finding of this study is the complete absence of any protective effect associated with Halal certification. With an identical detection rate of 29.2% in both Halal-certified and non-certified products (OR = 1, *p* = 1), the data demonstrate operational vulnerabilities in current voluntary Halal certification procedures in the absence of mandatory molecular screening. These data highlight the necessity of integrating routine qPCR testing into audit protocols from both a food safety and a religious compliance perspective.

It is important to distinguish between food fraud (economically motivated intentional substitution), regulatory non-compliance (failure to meet labelling standards), and Halal non-compliance (presence of prohibited porcine material regardless of intent). Molecular detection alone can identify non-compliance and high DNA abundance but cannot definitively prove fraudulent intent.

Several factors may explain this paradox. First, the Halal certification landscape in Kazakhstan is characterised by fragmentation, with multiple certification bodies operating without standardised audit protocols or mutual recognition agreements. Unlike countries such as Malaysia or Indonesia, where centralised Halal certification authorities (JAKIM and BPJPH, respectively) enforce rigorous and standardised certification processes [29,30,31], Kazakhstan’s framework remains fragmented. This fragmentation creates opportunities for certification bodies with inadequate auditing procedures to issue Halal labels to facilities that do not meet the required standards.

Second, the possibility of fraudulent or counterfeit Halal certificates cannot be dismissed. In the absence of a centralised digital registry or verification system, it is relatively easy for manufacturers to display Halal labels on their products without having undergone legitimate certification. Third, even genuinely certified facilities may experience adulteration through shared production lines. Many meat processing plants in Kazakhstan produce both Halal and non-Halal products on the same equipment, and the adequacy of cleaning and changeover protocols between production runs is not routinely verified by external auditors.

The finding that 18 of 19 positive Halal-certified samples had Ct < 35 is of particular concern. This indicates that porcine DNA was present in substantial, not trace, quantities in Halal-labelled products, which is inconsistent with a cross-contamination explanation and strongly suggests either non-compliant raw material usage at the manufacturing facility or the use of non-certified raw materials (including pork) in the production process. Either scenario represents a fundamental failure of the Halal assurance system.

The borderline association between high-level processing and porcine DNA detection (33.1% vs. 18.9%, *p* = 0.0773) aligns with the broader food fraud literature, which consistently identifies comminuted and highly processed meat products as the most vulnerable to species substitution [32,33]. Processing methods [34] such as emulsification, thermal treatment, and smoking alter the visual, textural, and organoleptic properties of meat, making it difficult or impossible for consumers and even trained inspectors to detect the presence of substituted species. The odds ratio of 2.127 suggests that highly processed products may be approximately twice as likely to contain undeclared porcine DNA as minimally processed products, a finding that, while not statistically significant at the conventional α = 0.05 level, is consistent with international trends and warrants confirmation in larger studies.

Several limitations should be acknowledged. First, the study used convenience sampling from retail outlets, which may not fully represent national product distribution. Second, the Ct threshold of 35, while informed by the qPCR literature [18,19,20], represents a pragmatic surveillance cut-off rather than a matrix-specific validated threshold, and future studies employing absolute quantification [35,36] with standard curves would provide more precise estimates of the proportion of pork tissue in positive samples. Third, a single target gene (mitochondrial cyt b) was used; confirmation with additional species-specific markers would strengthen the specificity of the findings. Fourth, DNA input was normalised by extraction mass and volume rather than by strictly adjusting total DNA concentration across samples; while this is standard practice for qualitative and semi-quantitative food screening to avoid diluting trace targets, variable total DNA input may introduce minor variance in absolute Ct values. Finally, the study did not investigate the point in the supply chain at which adulteration occurred (raw material supply, processing, or distribution), which would be valuable for designing targeted interventions. Additionally, although a run-level positive control was included, the absence of a sample-specific internal amplification control (IAC) means that partial PCR inhibition in individual sample extracts cannot be entirely ruled out. For future work involving the analysis of real food samples such as meat, we strongly recommend incorporating an IAC or a positive amplification control in each reaction to definitively confirm the absence of PCR inhibitors and prevent potential false-negative results.

## 5. Conclusions

This study provides the first molecular evidence that undeclared porcine DNA is highly prevalent (29.2%) in processed meat products available to consumers in Kazakhstan. The finding that Halal certification offers no protective effect against porcine contamination, with identical detection rates in certified and non-certified products, highlights the need for institutional strengthening of the Halal certification framework in the country. The predominance of low Ct values (Ct < 35) points to the intake of raw materials containing undeclared components, emphasising the importance of supply chain standardisation.

Based on these findings, the following recommendations are proposed for Kazakhstani food safety authorities: (1) implementation of routine, randomised molecular surveillance programs for species authentication in processed meat products; (2) mandatory adoption of HACCP protocols with specific critical control points for species segregation and equipment sanitation in all meat processing facilities; (3) introduction of a digital Halal certificate verification system [37] accessible to consumers; and (4) enforcement of administrative accountability for label non-compliance to ensure consumer protection and serve as an effective deterrent. These measures are essential not only for protecting consumer rights and religious freedoms but also for maintaining the integrity of Kazakhstan’s domestic food supply and its potential as a future exporter of Halal-certified products to international markets.

## Figures and Tables

**Table 1:** Association between processing level and porcine DNA detection.

Processing Level	Positive	Negative	Total	Prevalence (%)
High	47	95	142	33.1
Low	10	43	53	18.9
Total	57	138	195	29.2

## Data Availability

Data underlying the analysis are available from the corresponding author on reasonable request, subject to Kazakhstan national regulations on the protection of commercial information.

## Supplementary Materials

The following supporting information can be downloaded at: https://www.mdpi.com/article/10.3390/foods15213876/s1, Table S1: Full dataset, *n* = 195.

## Abbreviations

The following abbreviations are used in this manuscript:

CI	Confidence Intervals
Ct	Cycle Threshold
DNA	Deoxyribonucleic Acid
ELISA	Enzyme-Linked Immunosorbent Assay
GMP	Good Manufacturing Practices
HACCP	Hazard Analysis Critical Control Point
NTC	No-Template Control
OR	Odds Ratios
PCR	Polymerase Chain Reaction
qPCR	Quantitative Polymerase Chain Reaction
SSOP	Standard Sanitation Operating Procedures
