# Supplementary material for: Molecular Detection of Undeclared Porcine DNA in Processed Meat Products and Assessment of Halal Labelling Compliance in Kazakhstan Using qPCR
Source: Foods. 2026 Oct 29;15(21):3876. doi: 10.3390/foods15213876 (PMC13763041; doi:10.3390/foods15213876)
Supplement: Supplementary file 1 [file foods-15-03876-s001.zip › foods-4546425-supplementary.pdf]

Table S1. Full dataset, *n*=195

Sample ID	Product Category	Halal Status	Processing Category	qPCR Result	Ct Value
TestProd_001	Horse	Unspecified	High	Positive	28.2
TestProd_002	Beef	Yes	High	Positive	26.8
TestProd_003	Beef	Yes	High	Negative	
TestProd_004	Horse	Yes	High	Negative	
TestProd_005	Beef	Yes	Low	Negative	
TestProd_006	Beef	Yes	High	Positive	31.05
TestProd_007	Beef	Yes	Low	Negative	
TestProd_008	Beef	Yes	High	Negative	
TestProd_009	Beef	Yes	High	Negative	
TestProd_010	Beef	Yes	High	Negative	
TestProd_011	Beef	Yes	High	Negative	
TestProd_012	Beef	Yes	High	Positive	28.63
TestProd_013	Beef	Yes	High	Negative	
TestProd_014	Beef	Yes	High	Negative	
TestProd_015	Horse	Yes	High	Positive	32.44
TestProd_016	Beef	Yes	High	Positive	27.42
TestProd_017	Horse	Yes	High	Positive	29.22
TestProd_018	Beef	Yes	High	Positive	26.03
TestProd_019	Beef	Unspecified	High	Negative	
TestProd_020	Horse	Unspecified	Low	Negative	
TestProd_021	Beef	Unspecified	Low	Negative	
TestProd_022	Beef	Yes	High	Negative	
TestProd_023	Beef	Yes	High	Negative	
TestProd_024	Horse	Yes	High	Positive	36.37
TestProd_025	Beef	Unspecified	High	Negative	
TestProd_026	Horse	Unspecified	High	Negative	
TestProd_027	Beef	Unspecified	High	Negative	
TestProd_028	Beef	Unspecified	High	Positive	35.88
TestProd_029	Beef	Unspecified	High	Negative	
TestProd_030	Beef	Unspecified	High	Positive	25.05
TestProd_031	Beef	Yes	High	Negative	
TestProd_032	Beef	Yes	High	Negative	
TestProd_033	Not specified	Unspecified	High	Positive	37.03
TestProd_034	Beef	Yes	High	Negative	
TestProd_035	Beef	Unspecified	High	Positive	31.07
TestProd_036	Beef	Unspecified	High	Positive	25.01
TestProd_037	Turkey	Unspecified	High	Negative	
TestProd_038	Turkey	Unspecified	High	Positive	30.02
TestProd_039	Beef	Unspecified	High	Positive	23.35

TestProd_040	Chicken	Unspecified	High	Positive	33.35
TestProd_041	Horse	Unspecified	Low	Negative	
TestProd_042	Horse	Unspecified	Low	Negative	
TestProd_043	Beef	Unspecified	Low	Negative	
TestProd_044	Turkey	Unspecified	Low	Negative	
TestProd_045	Turkey	Unspecified	High	Negative	
TestProd_046	Cheese-based	Unspecified	High	Negative	
TestProd_047	Beef	Unspecified	High	Positive	28.06
TestProd_048	Beef	Unspecified	High	Negative	
TestProd_049	Beef	Unspecified	Low	Negative	
TestProd_050	Mutton	Unspecified	Low	Negative	
TestProd_051	Beef	Unspecified	High	Negative	
TestProd_052	Chicken	Unspecified	High	Positive	29.07
TestProd_053	Mutton	Unspecified	Low	Negative	
TestProd_054	Horse	Unspecified	Low	Negative	
TestProd_055	Beef	Unspecified	High	Negative	
TestProd_056	Horse	Unspecified	High	Negative	
TestProd_057	Horse	Unspecified	High	Negative	
TestProd_058	Turkey	Unspecified	Low	Negative	
TestProd_059	Beef	Unspecified	Low	Negative	
TestProd_060	Beef	Unspecified	Low	Positive	37.01
TestProd_061	Mutton	Unspecified	Low	Negative	
TestProd_062	Chicken	Unspecified	Low	Negative	
TestProd_063	Beef	Yes	High	Negative	
TestProd_064	Beef	Yes	Low	Negative	
TestProd_065	Horse	Unspecified	High	Positive	31
TestProd_066	Chicken	Unspecified	Low	Negative	
TestProd_067	Beef	Unspecified	Low	Negative	
TestProd_068	Mutton	Unspecified	Low	Negative	
TestProd_069	Horse	Unspecified	Low	Negative	
TestProd_070	Beef	Unspecified	High	Negative	
TestProd_071	Beef	Unspecified	High	Negative	
TestProd_072	Turkey	Unspecified	High	Negative	
TestProd_073	Beef	Yes	High	Negative	
TestProd_074	Beef	Unspecified	High	Positive	24.04
TestProd_075	Beef	Unspecified	High	Negative	
TestProd_076	Beef	Unspecified	Low	Negative	
TestProd_077	Mutton	Unspecified	Low	Negative	
TestProd_078	Horse	Yes	High	Negative	
TestProd_079	Horse	Yes	High	Negative	
TestProd_080	Beef	Unspecified	High	Positive	23.76
TestProd_081	Chicken	Unspecified	Low	Negative	

TestProd_082	Chicken	Unspecified	High	Positive	21.22
TestProd_083	Beef	Unspecified	Low	Negative	
TestProd_084	Beef	Unspecified	Low	Negative	
TestProd_085	Turkey	Unspecified	High	Negative	
TestProd_086	Beef	Unspecified	High	Negative	
TestProd_087	Not specified	Unspecified	Low	Positive	23.04
TestProd_088	Turkey	Unspecified	Low	Negative	
TestProd_089	Beef	Unspecified	High	Negative	
TestProd_090	Not specified	Unspecified	High	Positive	13.65
TestProd_091	Beef	Unspecified	High	Negative	
TestProd_092	Horse	Unspecified	High	Negative	
TestProd_093	Beef	Yes	High	Negative	
TestProd_094	Beef	Unspecified	High	Positive	36.66
TestProd_095	Beef	Unspecified	High	Negative	
TestProd_096	Beef	Yes	High	Negative	
TestProd_097	Beef	Unspecified	High	Negative	
TestProd_098	Beef	Unspecified	High	Positive	24.04
TestProd_099	Horse	Yes	High	Negative	
TestProd_100	Beef	Yes	High	Negative	
TestProd_101	Beef	Unspecified	High	Positive	37.65
TestProd_102	Horse	Unspecified	High	Negative	
TestProd_103	Beef	Yes	High	Negative	
TestProd_104	Beef	Unspecified	High	Positive	37.04
TestProd_105	Beef	Unspecified	High	Negative	
TestProd_106	Beef	Unspecified	High	Negative	
TestProd_107	Beef	Yes	High	Negative	
TestProd_108	Beef	Unspecified	High	Negative	
TestProd_109	Beef	Unspecified	High	Positive	27.08
TestProd_110	Beef	Unspecified	High	Positive	25.04
TestProd_111	Beef	Unspecified	High	Positive	25.89
TestProd_112	Beef	Unspecified	High	Negative	
TestProd_113	Beef	Unspecified	High	Negative	
TestProd_114	Beef	Yes	High	Negative	
TestProd_115	Beef	Yes	High	Negative	
TestProd_116	Horse	Yes	High	Negative	
TestProd_117	Beef	Unspecified	High	Positive	28.68
TestProd_118	Beef	Unspecified	High	Positive	29.34
TestProd_119	Beef	Unspecified	High	Positive	27.96
TestProd_120	Turkey	Unspecified	High	Negative	
TestProd_121	Turkey	Unspecified	High	Negative	
TestProd_122	Chicken	Unspecified	Low	Negative	
TestProd_123	Not specified	Unspecified	High	Negative	

TestProd_124	Beef	Unspecified	High	Positive	36.92
TestProd_125	Chicken	Unspecified	High	Negative	
TestProd_126	Chicken	Unspecified	High	Negative	
TestProd_127	Beef	Unspecified	High	Negative	
TestProd_128	Beef	Unspecified	High	Negative	
TestProd_129	Beef	Unspecified	High	Negative	
TestProd_130	Chicken	Unspecified	High	Negative	
TestProd_131	Horse	Unspecified	High	Negative	
TestProd_132	Beef	Unspecified	High	Negative	
TestProd_133	Beef	Unspecified	High	Negative	
TestProd_134	Beef	Unspecified	High	Positive	24.03
TestProd_135	Beef	Unspecified	High	Negative	
TestProd_136	Beef	Unspecified	High	Negative	
TestProd_137	Not specified	Unspecified	Low	Negative	
TestProd_138	Beef	Unspecified	Low	Positive	26.08
TestProd_139	Beef	Unspecified	High	Negative	
TestProd_140	Beef	Unspecified	High	Negative	
TestProd_141	Beef	Unspecified	High	Negative	
TestProd_142	Beef	Unspecified	High	Negative	
TestProd_143	Cheese-based	Unspecified	High	Negative	
TestProd_144	Beef	Unspecified	High	Negative	
TestProd_145	Beef	Yes	Low	Positive	27.12
TestProd_146	Beef	Yes	Low	Negative	
TestProd_147	Beef	Yes	Low	Negative	
TestProd_148	Beef	Yes	Low	Negative	
TestProd_149	Beef	Yes	Low	Positive	28.04
TestProd_150	Beef	Yes	Low	Positive	26.18
TestProd_151	Beef	Yes	Low	Positive	29.16
TestProd_152	Beef	Yes	Low	Positive	28
TestProd_153	Beef	Yes	Low	Positive	32.01
TestProd_154	Beef	Yes	Low	Positive	29.01
TestProd_155	Beef	Unspecified	Low	Negative	
TestProd_156	Beef	Yes	Low	Negative	
TestProd_157	Beef	Unspecified	High	Negative	
TestProd_158	Horse	Yes	High	Negative	
TestProd_159	Horse	Yes	High	Negative	
TestProd_160	Chicken	Unspecified	High	Negative	
TestProd_161	Turkey	Unspecified	High	Negative	
TestProd_162	Chicken	Yes	High	Negative	
TestProd_163	Chicken	Yes	Low	Negative	
TestProd_164	Beef	Yes	High	Negative	
TestProd_165	Beef	Yes	High	Negative	

TestProd_166	Beef	Yes	High	Positive	26.03
TestProd_167	Beef	Yes	High	Positive	25.85
TestProd_168	Beef	Yes	High	Positive	27.01
TestProd_169	Beef	Yes	High	Positive	27.16
TestProd_170	Beef	Yes	High	Negative	
TestProd_171	Mutton	Unspecified	Low	Negative	
TestProd_172	Beef	Unspecified	Low	Negative	
TestProd_173	Chicken	Unspecified	Low	Negative	
TestProd_174	Beef	Unspecified	Low	Negative	
TestProd_175	Beef	Unspecified	Low	Negative	
TestProd_176	Horse	Yes	High	Negative	
TestProd_177	Beef	Yes	High	Negative	
TestProd_178	Turkey	Unspecified	High	Positive	37.08
TestProd_179	Beef	Unspecified	Low	Negative	
TestProd_180	Turkey	Unspecified	Low	Negative	
TestProd_181	Beef	Unspecified	High	Negative	
TestProd_182	Beef	Unspecified	High	Negative	
TestProd_183	Turkey	Unspecified	High	Negative	
TestProd_184	Beef	Yes	High	Negative	
TestProd_185	Chicken	Yes	High	Negative	
TestProd_186	Beef	Unspecified	High	Positive	26.17
TestProd_187	Chicken	Yes	High	Negative	
TestProd_188	Beef	Yes	High	Negative	
TestProd_189	Horse	Unspecified	High	Positive	31
TestProd_190	Chicken	Unspecified	High	Positive	18.91
TestProd_191	Beef	Unspecified	High	Positive	22.33
TestProd_192	Beef	Unspecified	High	Positive	28.12
TestProd_193	Turkey	Unspecified	Low	Negative	
TestProd_194	Beef	Unspecified	High	Positive	27.08
TestProd_195	Beef	Unspecified	High	Negative	
